# Predictability of maxillary transverse changes with Invisalign in adults: A systematic review and meta-analysis

**DOI:** 10.1097/MD.0000000000049772

**Published:** 2026-07-24

**Authors:** Hou XiangGang, Qin Jia, Yang Jie, Cao Yani, Liu Chao, Jincai Guo, Minghai Bai

**Affiliations:** a School of Stomatology, Hunan University of Traditional Chinese Medicine, Changsha, Hunan Province, China; b Department of Orthodontics, Changsha Stomatological Hospital, Changsha, Hunan Province, China; c Department of Pharmacy, Changsha Stomatological Hospital, Changsha, Hunan Province, China.

**Keywords:** arch expansion, Invisalign, meta-analysis, predictability, systematic review

## Abstract

**Background:**

To systematically evaluate the predictability of Invisalign for maxillary transverse expansion in adult patients.

**Methods:**

Five databases – PubMed, Embase, Scopus, Web of Science, and the Cochrane Library – were searched for clinical studies published between January 1, 2014, and August 1, 2024, that evaluated Invisalign for maxillary transverse expansion. After study selection and data extraction, meta-analyses were performed using Stata/MP, version 17.0 (StataCorp LLC).

**Results:**

Six retrospective clinical studies involving 233 patients were eligible for inclusion. The meta-analysis showed varying degrees of predictability across different regions of the maxilla treated with Invisalign. Specifically, the pooled predictability was 72.01% in the canine region (95% confidence interval [CI]: 59.67%–84.35%; *P* = .001; *I*^2^ = 74.7%), 80.73% in the 1st premolar region (95% CI: 73.78%–87.68%; *P* = .115; *I*^2^ = 43.5%), 78.74% in the 2nd premolar region (95% CI: 70.41%–87.08%; *P* = .039; *I*^2^ = 57.4%), and 71.57% in the 1st molar region (95% CI: 62.41%–80.73%; *P* = .074; *I*^2^ = 50.2%). Studies conducted in developed countries showed a higher pooled predictability (77.22%; 95% CI: 70.34%–84.10%; *P* = .454; *I*^2^ = 0.0%) than those conducted in developing countries (58.53%; 95% CI: 45.20%–71.86%; *P* = .225; *I*^2^ = 32.0%), with a statistically significant difference between the subgroups (*P* = .015). Studies using Geomagic software (3D Systems) for measurements generally showed lower heterogeneity.

**Conclusion:**

The available evidence suggests that Invisalign provides moderate predictability for maxillary transverse expansion, with the highest pooled predictability in the premolar regions and lower predictability in the canine and 1st molar regions. The canine estimate should be interpreted cautiously because of substantial heterogeneity and low-certainty evidence. Exploratory subgroup findings related to study-country classification, anatomical landmarks, and measurement software remain uncertain because of the small number of studies and methodological variability. Further well-designed prospective studies are needed to confirm these findings.

## 1. Introduction

Maxillary transverse expansion techniques have been widely used to correct dental arch crowding and posterior crossbites, enhance smile aesthetics, and compensate for maxillary hypoplasia, among other indications.^[1–3]^ The fundamental mechanisms by which these techniques achieve maxillary expansion differ and can be broadly categorized into 4 types: tooth movement, tooth tilting, dentoalveolar bending and tilting, and midpalatal suture opening. In recent years, clear aligner therapy, exemplified by Invisalign, has gained increasing favor among patients over traditional fixed appliances because of its aesthetic and comfort advantages.^[4,5]^ Invisalign reportedly facilitates gradual maxillary transverse expansion through buccal tipping and bodily movement of the teeth. It may serve as an alternative to interproximal reduction by creating space to align the dentition and address problems such as crowding or crossbites.^[6–8]^

Invisalign employs a series of precisely fitted clear aligners to incrementally reposition misaligned teeth. The production of these aligners integrates computer-aided design and computer-aided manufacturing with stereolithography technology and allows the treatment process and predicted outcomes to be visualized using ClinCheck software (Align Technology, Inc.).^[9]^ Despite the potential for precise tooth movement, clinical treatment outcomes do not always fully correspond to those predicted by the software.^[10]^ In clinical practice, many cases still require mid-course corrections, refinements, or conversion to fixed appliances before treatment is completed. The predictability of treatment outcomes is therefore an important factor contributing to the success of orthodontic interventions.^[11]^ Consequently, numerous studies have been conducted in recent years to assess the predictability of clear aligners. By quantifying predictability, clinicians may identify dimensions in which the final tooth position deviates from the predicted position, allowing necessary compensations to be incorporated into the virtual treatment plan. Several studies have investigated the application of clear aligners for maxillary expansion, but their findings have been inconsistent.^[6,8,12–15]^ The present systematic review and meta-analysis aims to evaluate the available evidence regarding the predictability of clear aligner therapy for arch expansion. Understanding the accuracy of software-predicted changes may help practitioners anticipate the need for overcorrection, thereby potentially reducing refinements, mid-course corrections, and overall treatment time.

## 2. Materials and methods

### 2.1. Protocol registration

The protocol for this systematic review and meta-analysis was registered in the International Prospective Register of Systematic Reviews (registration number CRD42024584778). This registration promotes transparency and adherence to the predefined methodology of the systematic review.

This study was based exclusively on previously published literature. No new data were collected directly from human participants, and no interventions were performed. Therefore, ethics committee or institutional review board approval was not required, and informed consent was not applicable.

### 2.2. Eligibility and exclusion criteria

Study eligibility was defined according to the Participants, Intervention, Comparator, Outcomes, and Study design framework. The detailed eligibility and exclusion criteria are presented in Table 1.

**Table 1:** Eligibility and exclusion criteria based on the PICOS framework.

PICOS component	Eligibility criteria	Exclusion criteria
Participants (P)	Studies involving patients with malocclusion requiring maxillary transverse expansion.	Studies involving patients with malocclusion associated with cleft lip and palate or other craniofacial syndromes.
Intervention (I)	Invisalign treatment, including the use of Invisalign attachments.	Studies using additional auxiliary devices, extraction protocols, surgical treatment, or other treatment modalities in combination with Invisalign.
Comparator (C)	Maxillary transverse expansion predicted using ClinCheck software.	Studies without clearly specified predicted values for comparison.
Outcomes (O)	The percentage of achieved maxillary transverse expansion relative to that predicted using ClinCheck software.	Studies lacking relevant outcome data.
Study design (S)	Randomized controlled trials, controlled clinical trials, single-arm clinical trials, cohort studies, and other eligible non-randomized clinical studies.	Case reports, reviews, laboratory studies, and 3-dimensional finite element analyses.

PICOS = Participants, Intervention, Comparator, Outcomes, and Study design.

### 2.3. Search methods

A systematic literature search was conducted in PubMed, Embase, Scopus, Web of Science, and the Cochrane Library for studies published between January 1, 2014, and August 1, 2024. The search strategy combined terms related to maxillary expansion and clear aligner therapy. The detailed PubMed search strategy is presented in Table 2 and was adapted as appropriate for the other databases.

**Table 2:** PubMed search strategy.

Steps	Strategies
1	“Palatal Expansion Technique”[Mesh]
2	((((Maxillary Expansion[Title/Abstract]) OR (dentoalveolar expansion[Title/Abstract])) OR (Palatal Expansion Technic*[Title/Abstract])) OR (transverse expansion[Title/Abstract])) OR (arch expansion[Title/Abstract])
3	1 OR 2
4	((((Removable Orthodontic Appliance*[Title/Abstract]) OR (Clear Aligner*[Title/Abstract])) OR (Clear Dental Brace*[Title/Abstract])) OR (Invisalign[Title/Abstract])) OR (transparent aligner*[Title/Abstract])
5	3 AND 4

Retrieved records were checked for duplicates before screening. Two reviewers independently screened the records, and any disagreements were resolved through discussion and consensus.

### 2.4. Study selection and data extraction

Two reviewers independently screened the titles and abstracts of the retrieved records according to the predefined eligibility criteria. The full texts of potentially eligible articles were subsequently assessed to determine final inclusion. The screening results were cross-checked, and any disagreements were resolved through discussion with a 3rd reviewer.

The following information was extracted from each included study: 1st author, publication year, country, sample size, participant age, study design, aligner replacement interval, follow-up duration, anatomical measurement site, measurement software, and the percentage of achieved maxillary transverse expansion relative to the expansion predicted using ClinCheck software.

### 2.5. Risk-of-bias assessment and certainty of evidence

The risk of bias in the included non-randomized studies was assessed using the Risk of Bias in Non-randomized Studies of Interventions (ROBINS-I) tool.^[16]^ The ROBINS-I assessment included bias due to confounding, participant selection, classification of interventions, deviations from intended interventions, missing data, outcome measurement, and selection of the reported results. The certainty of evidence for each outcome was evaluated using the Grading of Recommendations Assessment, Development and Evaluation approach and classified as high, moderate, low, or very low.^[17]^ Two reviewers independently performed the assessments. Any disagreements were resolved through discussion or consultation with a 3rd reviewer.

### 2.6. Data analysis

Meta-analyses were performed using Stata/MP, version 17.0 (StataCorp LLC). Predictability was defined as the percentage of achieved maxillary transverse expansion relative to the expansion predicted using ClinCheck software. Because clinical and methodological heterogeneity was anticipated across studies, the primary pooled estimates were calculated using DerSimonian–Laird random-effects models and reported with corresponding 95% confidence intervals (CIs). Statistical heterogeneity was assessed using Cochran *Q* test and the *I*^2^ statistic, with *I*^2^ values of approximately 25%, 50%, and 75% interpreted as low, moderate, and substantial heterogeneity, respectively. Exploratory subgroup analyses were conducted using inverse-variance methods according to anatomical measurement landmark, measurement software, and study-country classification. Additional potential sources of heterogeneity, including programmed expansion magnitude, aligner replacement protocols, attachment design and distribution, clinician experience, software version, treatment stage, and patient adherence, were also considered. However, these variables were inconsistently or incompletely reported across the included studies and therefore could not be assessed quantitatively. The country-based classification was used only as an exploratory study-level grouping and was not intended to represent individual socioeconomic status. Leave-one-out sensitivity analyses were performed by sequentially excluding individual studies. Because fewer than 10 studies were available for each outcome, funnel plots were not considered sufficiently reliable; Egger regression test was used only as an exploratory assessment of small-study effects. Statistical significance was set at α = 0.05. All subgroup and small-study-effect findings were interpreted cautiously because of the limited number of included studies.

## 3. Results

### 3.1. Literature search and study characteristics

The systematic search strategy yielded a total of 269 articles. After excluding duplicates, 126 articles remained. Following the initial screening based on titles and abstracts, 111 articles were excluded as they did not meet the inclusion criteria. Upon obtaining and reviewing the full texts, an additional 9 articles were excluded. Ultimately, 6 studies were included in the final analysis^[6,8,12–15]^ (Preferred Reporting Items for Systematic Reviews and Meta-Analyses flow diagram).

The 6 included studies comprised a total of 233 patients treated with Invisalign for malocclusion requiring maxillary transverse expansion. Among the studies that reported participant sex, 117 participants were female and 59 were male; sex was not reported for the remaining participants. The characteristics of the included studies are presented in Table 3.

**Table 3:** Characteristics of included studies.

Author, year, country	Study design	Population			Intervention	Comparison	Measurement				Outcome
		Sample size, n	Sex	Age			Landmark			Software	
Houle et al, 2017, Australia	Retrospective study	64	41F 23M	18–61 yr MA: 31.2 yr	①; ②	③	Palatal cusp tips		Geomagic Qualify software		④ of ICW, FIPW, SIPW and FIMW.
Zhao et al, 2017, China	Retrospective study	31	24 F 7M	18–32 yr MA: 24.1 ± 4 yr	①; ②	③	Central fossa		3-Matic 8.0		④ of ICW, FIPW, SIPW, FIMW and SIMW.
Zhou et al, 2020, China	Retrospective study	20	15F 5M	20–45 yr MA: 28.5 ± 6.3 yr	①; ②	③	Palatal cusp tips		Geomagic Studio 12.0 software		④ of ICW, FIPW, SIPW and FIMW.
Lione et al, 2021, Italy	Retrospective study	28	16F 12M	MA: 31.9 ± 5.4 yr	①; ②	③	Buccal cusp tips	Viewbox 4 software			⑤ of ICW, FIPW, SIPW, FIMW and SIMW.
Bowman et al, 2023, Australia	Retrospective study	33	21F 12M	Over 18 yr MA: 32.7 ± 13.12 yr	①; ②	③	Buccal cusp tips	Geomagic Control X software			⑤ of ICW, FIPW, SIPW, FIMW and SIMW.
Tien et al, 2023, Australia	Retrospective study	57	–	Over 20 yr	①; ②	③	Buccal cusp tips	MeshLab			④ of ICW, FIPW, SIPW, FIMW and SIMW.

F = female, FIMW = 1st intermolar width, FIPW = 1st interpremolar width, ICW = intercanine width, M = male, SIMW = 2nd intermolar width, SIPW = 2nd interpremolar width.

① Invisalign treatment with attachments, without additional auxiliary devices, extraction protocols, surgical treatment, or other treatment modalities.

② Quantitative measurements of predicted and achieved maxillary transverse expansion.

③ Tooth-movement predictions generated using ClinCheck software.

④ Predictability of changes in maxillary transverse measurements.

⑤ Statistical comparison between the achieved posttreatment measurements and the posttreatment measurements predicted using ClinCheck.

### 3.2. Risk of bias in the included studies

All 6 included studies were non-randomized and were therefore assessed using the ROBINS-I tool. Two studies were judged to have an overall low risk of bias,^[8,13]^ whereas 4 studies were judged to have an overall moderate risk of bias.^[6,12,14,15]^ The main concerns were related to confounding, participant selection, classification of interventions, outcome measurement, and selection of reported results. The detailed ROBINS-I assessments are presented in Table 4.

**Table 4:** Risk-of-bias assessment of the included studies using the ROBINS-I tool.

Article reference	Houle^[8]^ 2017	Zhao^[12]^ 2017	Zhou^[6]^ 2020	Lione^[13]^ 2021	Bowman^[14]^ 2023	Tien^[15]^ 2023
Bias due to confounding	L	M	M	L	M	M
Bias in the selection of participants for the study	L	M	M	L	M	L
Bias in the classification of interventions	L	L	M	L	L	L
Bias due to deviations from intended interventions	L	L	L	L	L	L
Bias due to missing data	L	L	L	L	L	L
Bias in the measurement of outcomes	L	L	M	L	M	L
Bias in the selection of the reported result	L	L	L	L	L	M
The overall risk of bias	L	M	M	L	M	M

L = low risk, M = moderate risk, ROBINS-I = Risk of Bias in Non-randomized Studies of Interventions.

### 3.3. Pooled predictability of maxillary transverse expansion

Six studies reported data on the predictability of Invisalign for maxillary transverse expansion by comparing the achieved expansion with the expansion predicted using ClinCheck software. Predictability was calculated as the percentage of achieved expansion relative to the predicted expansion. A total of 233 patients were included in the quantitative synthesis.

- Canine region: The pooled predictability was 72.01% (95% CI: 59.67%–84.35%), with substantial heterogeneity (*P* = .001; *I*^2^ = 74.7%).
- First premolar region: The pooled predictability was 80.73% (95% CI: 73.78%–87.68%), with low-to-moderate heterogeneity (*P* = .115; *I*^2^ = 43.5%).
- Second premolar region: The pooled predictability was 78.74% (95% CI: 70.41%–87.08%), with moderate heterogeneity (*P* = .039; *I*^2^ = 57.4%).
- First molar region: The pooled predictability was 71.57% (95% CI: 62.41%–80.73%), with moderate heterogeneity (*P* = .074; *I*^2^ = 50.2%).

**Figure 1.**
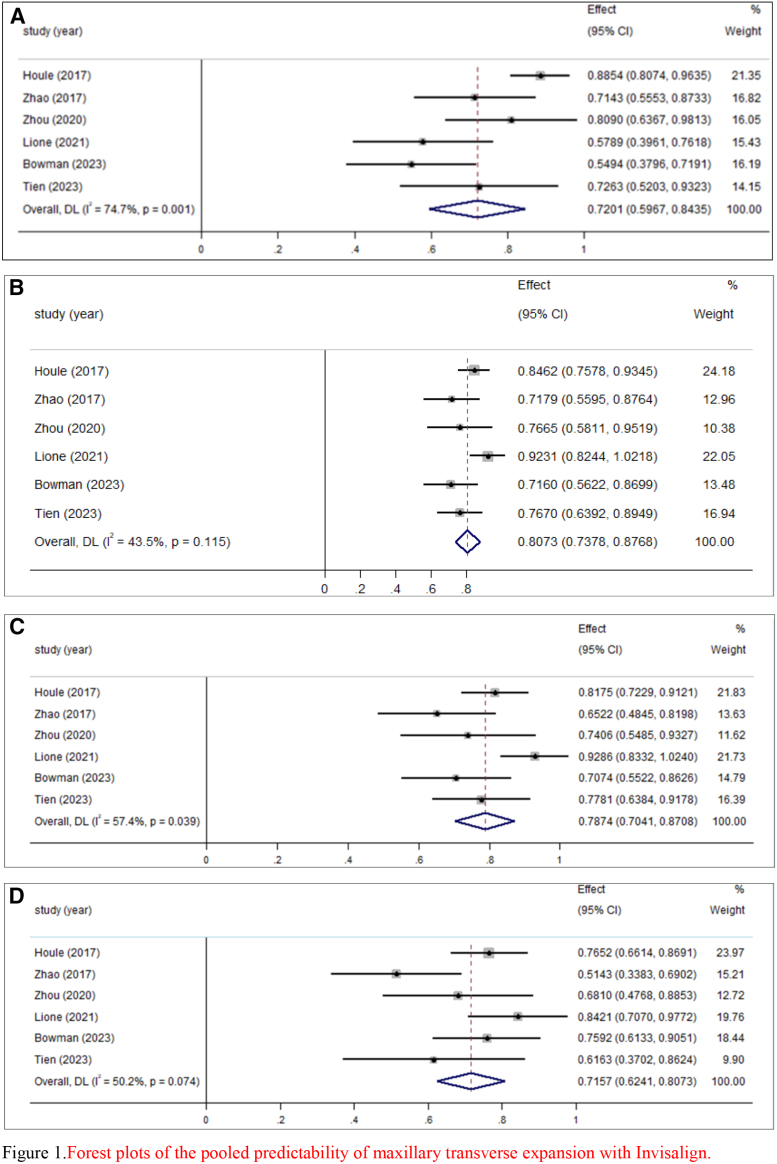
Forest plots of the pooled predictability of maxillary transverse expansion with Invisalign. (A) Canine region; (B) 1st premolar region; (C) 2nd premolar region; and (D) 1st molar region. CI = confidence interval, DL = DerSimonian-Laird.

Predictability was higher in the premolar regions than in the canine and 1st molar regions. The corresponding forest plots are presented in Figure 1.

### 3.4. Subgroup analyses

**Figure 2.**
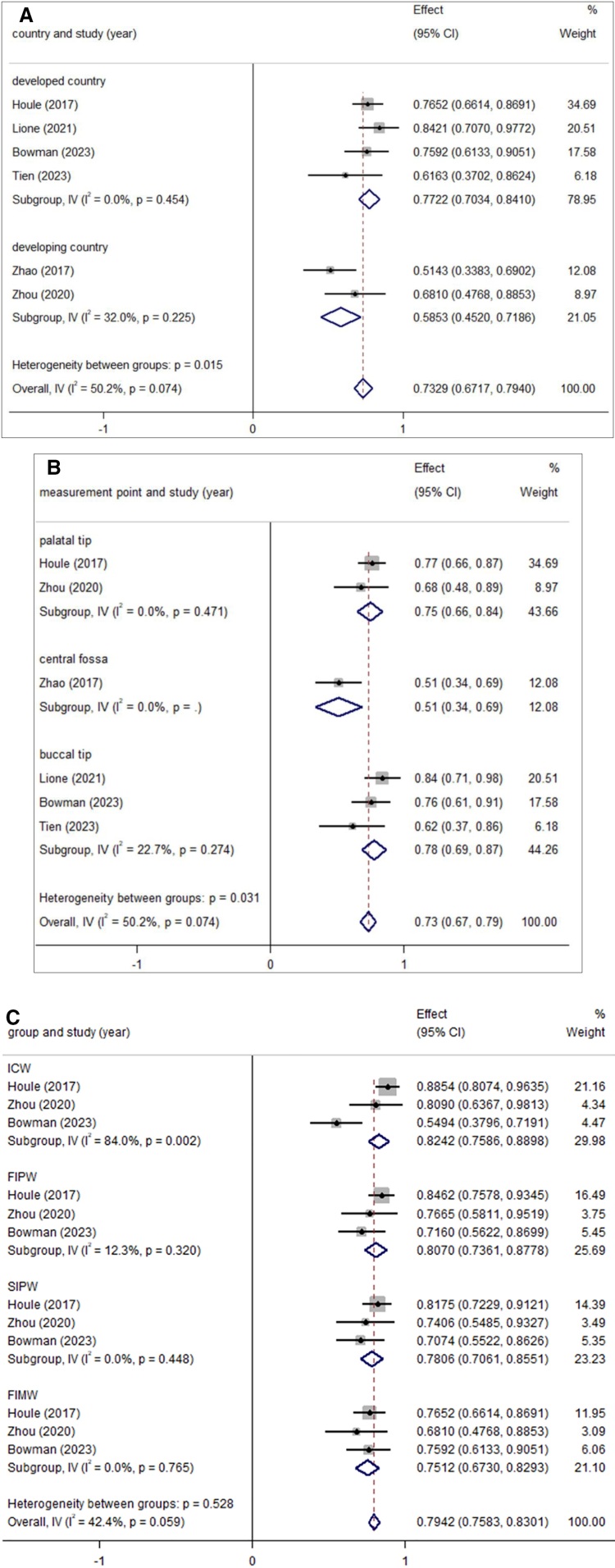
Subgroup analyses of the predictability of maxillary transverse expansion. (A) First molar region stratified by the country classification of the included studies; (B) 1st molar region stratified by the anatomical measurement landmark; and (C) pooled predictability estimates for the canine, 1st premolar, 2nd premolar, and 1st molar regions among studies using Geomagic software. CI = confidence interval.

Exploratory subgroup analyses were conducted according to the country classification of the included studies, anatomical measurement landmarks, and the use of Geomagic software. For the 1st molar region, studies conducted in developed countries showed a pooled predictability of 77.22% (95% CI: 70.34%–84.10%), with no observed heterogeneity (*P* = .454; *I*^2^ = 0.0%). In contrast, studies conducted in developing countries showed a pooled predictability of 58.53% (95% CI: 45.20%–71.86%), with low heterogeneity (*P* = .225; *I*^2^ = 32.0%). The difference between the 2 subgroups was statistically significant (*P* for subgroup difference = .015) (Fig. 2A). However, because only 2 studies were included in the developing-country subgroup, this finding should be interpreted cautiously. This country-based classification was used only as an exploratory study-level grouping and should not be interpreted as a measure of individual socioeconomic status or patient behavior.

Subgroup analysis according to the anatomical measurement landmark also showed a statistically significant difference among the palatal cusp-tip, central-fossa, and buccal cusp-tip subgroups in the 1st molar region (*P* for subgroup difference = .031) (Fig. 2B). The pooled predictability was 75% (95% CI: 66%–84%) for studies using palatal cusp tips and 78% (95% CI: 69%–87%) for studies using buccal cusp tips. The study using the central fossa reported a lower predictability of 51% (95% CI: 34%–69%). After excluding the central-fossa subgroup, no statistically significant difference was observed between the palatal and buccal cusp-tip subgroups (*P* = .655).

Among the 3 studies using Geomagic software, the pooled predictability estimates were 82.42% (95% CI: 75.86%–88.98%) for the canine region, 80.70% (95% CI: 73.61%–87.78%) for the 1st premolar region, 78.06% (95% CI: 70.61%–85.51%) for the 2nd premolar region, and 75.12% (95% CI, 67.30%–82.93%) for the 1st molar region (Fig. 2C). Heterogeneity was substantial in the canine region (*I*^2^ = 84.0%; *P* = .002), but low in the 1st premolar region (*I*^2^ = 12.3%; *P* = .320) and absent in the 2nd premolar and 1st molar regions (*I*^2^ = 0.0%; *P* = .448 and *P* = .765, respectively). No statistically significant difference was observed among the 4 anatomical regions within the Geomagic subgroup (*P* for subgroup difference = .528).

### 3.5. Sensitivity analysis

A leave-one-out sensitivity analysis was performed by sequentially excluding each study and recalculating the pooled estimates using the random-effects model. The pooled estimates for the premolar and 1st molar regions remained generally similar after the exclusion of individual studies, suggesting that these estimates were not driven by a single study. However, sensitivity analysis does not remove or explain between-study heterogeneity, particularly in the canine region.

### 3.6. Assessment of publication bias

Because fewer than 10 studies were included for each outcome, funnel plots were not considered sufficiently reliable for assessing publication bias. Egger regression test was therefore performed for outcomes reported by all 6 included studies. The results were as follows: 1st premolar region, *t* = −2.56 and *P* = .064; 2nd premolar region, *t* = −2.75 and *P* = .051; 1st molar region, *t* = −1.58 and *P* = .190; and canine region, *t* = −2.83 and *P* = .047.

At the prespecified significance level of 0.05, Egger test indicated potential small-study effects in the canine region but not in the 1st premolar, 2nd premolar, or 1st molar regions. However, because only 6 studies were included in each analysis, the statistical power of Egger test was limited, and these results should be interpreted cautiously.

### 3.7. Certainty of evidence

The certainty of evidence for the predictability of maxillary transverse expansion ranged from low to high across the evaluated regions. The certainty of evidence was rated as low for the intercanine region, high for the 1st interpremolar region, and moderate for the 2nd interpremolar and 1st intermolar regions.

The evidence for the intercanine region was downgraded because of suspected publication bias and substantial inconsistency among the included studies. The evidence for the 2nd interpremolar and 1st intermolar regions was downgraded because of concerns regarding risk of bias and moderate heterogeneity. The detailed Grading of Recommendations Assessment, Development and Evaluation assessment is presented in Table 5. These ratings indicate that additional high-quality studies may affect confidence in the pooled estimates, particularly for the intercanine, 2nd interpremolar, and 1st intermolar regions.

**Table 5:** Quality of the evidence according to GRADE.

Outcomes		No. of observations	Risk of bias	Inconsistency	Indirectness	Imprecision	Other considerations	Quality of the evidence (GRADE)	Effect size (%) (95% CI)
Maxillary intercanine	194		Serious	Serious	Not serious	Not serious	①②	⨁⨁◯◯ Low*^,^†	72.01% (59.67%–84.35%)
Maxillary 1st interpremolar	218		Not serious	Not serious	Not serious	Not serious	②	⨁⨁⨁⨁ High	80.73% (73.78%–87.68%)
Maxillary 2nd interpremolar	210		Serious	Serious	Not serious	Not serious	②	⨁⨁⨁◯ Moderate‡	78.74% (70.41%–87.08%)
Maxillary 1st intermolar	191		Serious	Serious	Not serious	Not serious	②	⨁⨁⨁◯ Moderate‡	71.57% (62.41%–80.73%)

CI = confidence interval, GRADE = Grading of Recommendations Assessment, Development and Evaluation.

* Due to publication bias.

† Effect sizes varied widely.

‡ Due to moderate heterogeneity.

① Publication bias strongly suspected.

② All plausible residual confounding would reduce the demonstrated effect.

## 4. Discussion

### 4.1. Advantages of clear aligner therapy over traditional orthodontic treatment

In contrast to traditional orthodontic treatment, which may require repeated clinical adjustments, clear aligner therapy is planned according to the desired final positions of the teeth. The amount and pathway of tooth movement can be designed before treatment, and the planned treatment process can be visualized using a reconstructed 3-dimensional model.^[18]^ This digitally guided and visually predictable treatment planning represents an important advantage of clear aligner therapy. The mode of force delivery depends on the design and mechanical properties of the orthodontic appliance. In removable polymeric aligners, force is generated by the geometric mismatch between the tooth surface and the aligner and is transferred through a relatively broad and less clearly defined contact area.^[19]^ For transverse expansion, each aligner is designed with a slightly greater arch width than the existing dental arch. When the aligner is inserted, elastic deformation generates forces that promote buccal tipping and bodily movement of the teeth, followed by remodeling of the alveolar bone and an increase in maxillary arch width.

### 4.2. Predictability of maxillary arch expansion

Individual patient characteristics, variations in aligner material properties, and the complexity of tooth movement may cause the achieved tooth displacement to differ from the programmed displacement.^[20]^ The present meta-analysis showed that the transverse expansion predicted using ClinCheck was not fully achieved in most regions. For each 1 mm of programmed expansion, approximately 0.72 to 0.81 mm was achieved across the canine, premolar, and 1st molar regions.

Predictability varied according to the anatomical region. The highest pooled predictability was observed in the 1st premolar region, followed by the 2nd premolar region, whereas lower predictability was observed in the canine and 1st molar regions. Differences in root morphology, buccal cortical bone thickness, soft-tissue resistance, periodontal ligament area, and masticatory loading may contribute to regional differences in expansion response.^[21–24]^ Posterior teeth generally have more complex root morphology and a larger periodontal ligament area, which may make controlled bodily movement more difficult. In addition, aligner control may decrease toward the posterior region, which may further reduce predictability.^[13]^

### 4.3. Clinical significance of predictability

Because the transverse expansion achieved with clear aligners may differ from the programmed values, accurate estimates of predictability are important for treatment planning and monitoring tooth movement. First, they may help orthodontists make informed treatment decisions and communicate realistic expectations and achievable outcomes to patients.^[25,26]^ Second, improved treatment predictability may reduce the need for additional refinements and treatment delays, thereby increasing the overall efficiency of orthodontic treatment.^[8,27]^ Finally, a better understanding of predictability may influence patients’ treatment choices, as treatments with more predictable outcomes may be perceived as more attractive.^[26,28]^ Clear aligner therapy also requires substantial patient cooperation. Therefore, treatment outcomes depend not only on the clinician’s skills and treatment-planning ability but also on patient compliance.^[29]^ Accurate prediction, regular monitoring, and timely communication with patients may contribute to improved treatment outcomes.

### 4.4. Implications for overcorrection

Understanding the accuracy of software-predicted tooth movement may help clinicians determine whether overcorrection is needed, potentially reducing refinements, mid-course corrections, and overall treatment time. Overcorrection involves programming movement beyond the desired final position to compensate for the expected difference between predicted and achieved tooth movement.^[30]^ Insufficient overcorrection may result in underexpansion and the need for additional treatment, whereas excessive overcorrection may increase the risk of adverse periodontal effects, such as bone dehiscence and fenestration.^[12]^ The pooled estimates in this meta-analysis indicate that achieved expansion was generally lower than programmed expansion, suggesting that some degree of additional programmed expansion may be considered in clinical planning. However, the present evidence does not support a universal numerical overcorrection prescription because of the limited number of studies, between-study heterogeneity, and individual variation in treatment response. In the studies by Zhou et al^[6]^ and Tien et al,^[15]^ predictability estimates exceeding 100% indicated that achieved expansion occasionally exceeded programmed expansion. This variability may be associated with attachment design and positioning, clinician-related treatment planning, software updates, aligner material properties, and patient adherence.^[8,9]^ Therefore, overcorrection should be individualized and adjusted according to the anatomical region, periodontal conditions, treatment response, and clinical monitoring. Further high-quality prospective studies are needed to establish evidence-based overcorrection protocols.

### 4.5. Heterogeneity in the canine region

The greatest between-study heterogeneity was observed in the canine region. Although the leave-one-out sensitivity analysis indicated that the pooled estimate was not disproportionately influenced by any single study, substantial heterogeneity remained. Moreover, the subgroup analyses according to study-country classification, measurement software, and anatomical measurement landmarks did not adequately explain the heterogeneity in this region. However, these findings do not exclude these factors as potential sources of heterogeneity because the subgroup analyses included only a small number of studies and therefore had limited statistical power. The anatomical position and root morphology of the maxillary canine may contribute to variability in treatment response. Rotational and transverse movements of the canine are particularly difficult to control using clear aligners. Other clinical and methodological factors may also have contributed to the residual heterogeneity, including programmed expansion magnitude, aligner replacement protocols, attachment design and distribution, clinician experience, software version, treatment stage, and patient adherence. Because these variables were inconsistently or incompletely reported in the primary studies, their effects could not be examined quantitatively. Charalampakis et al^[31]^ reported that rotational movement showed relatively low predictability, with the maxillary canine among the most affected teeth. Tommaso et al^[32]^ reported that approximately 0.4° of canine rotation was achieved for every 1° of programmed rotation. Therefore, the rotational component of canine movement should be considered when planning expansion and potential overcorrection in this region. Because the included studies did not provide sufficiently detailed data on the magnitude and direction of canine rotation, attachment configuration, root position, or movement pattern, these potential sources of heterogeneity could not be examined quantitatively. Given the importance of the maxillary canine to dental arch form and smile aesthetics,^[33]^ clinicians should carefully monitor this region during treatment. Future studies should report canine-specific movement characteristics in greater detail to clarify the factors influencing the predictability of transverse expansion.

### 4.6. Influence of anatomical landmark selection

The 6 included studies used different anatomical landmarks to measure maxillary transverse width, including buccal cusp tips, palatal cusp tips, and central fossae. Although each study used consistent landmarks when calculating the ratio of achieved expansion to ClinCheck-predicted expansion, differences in landmark selection may still introduce methodological heterogeneity.

Because clear aligner expansion is achieved through a combination of tooth tipping and bodily movement, landmarks located at different positions on the crown may capture different magnitudes of displacement. Buccal cusp tips may reflect a greater component of buccal tipping and have been associated with relatively high predictability,^[34]^ whereas measurements based on palatal cusp tips, lingual gingival margins, or central fossae may more closely reflect movement nearer the center of the crown or tooth.^[8]^ This explanation was partly supported by the exploratory subgroup analysis. In the 1st molar region, the study using the central fossa as the measurement landmark showed a lower actual-to-predicted ratio than studies using buccal or palatal cusp tips. In contrast, no statistically significant difference was observed between the buccal and palatal cusp-tip subgroups after the central-fossa study was excluded. These findings suggest that the measured predictability may be influenced more by whether the landmark is a cusp tip or an occlusal fossa than by its buccal or palatal position alone. However, because only 1 study used the central fossa and only 6 studies were included overall, this finding should be regarded as exploratory. Further studies using standardized anatomical landmarks are required to clarify the influence of landmark selection on measured expansion predictability.

### 4.7. Interpretation of subgroup analyses

The subgroup analysis showed that studies using Geomagic software had low or no heterogeneity in the 1st premolar, 2nd premolar, and 1st molar regions, although substantial heterogeneity remained in the canine region. Sousa et al^[35]^ previously demonstrated the accuracy and reproducibility of Geomagic software for linear dental measurements, suggesting that the use of a consistent measurement platform may contribute to more comparable results. However, because only 3 included studies used Geomagic software, it cannot be concluded that the software itself was responsible for the lower heterogeneity. A statistically significant difference in pooled predictability was also observed between studies conducted in developed and developing countries, with higher predictability reported in the former. This comparison was an exploratory study-level analysis, and only 2 studies were included in the developing-country subgroup. The country grouping was not based on individual-level socioeconomic, educational, behavioral, or compliance data. Therefore, the observed difference should not be attributed directly to national economic status, oral-health awareness, or patient compliance and may instead reflect multiple unmeasured differences among studies, including patient characteristics, clinician experience, treatment protocols, attachment design and distribution, aligner replacement schedules, software versions, and treatment stages.

The subgroup analyses suggest that measurement software, anatomical landmarks, and other study-level characteristics may contribute to the observed heterogeneity. Additional factors, including programmed expansion magnitude, aligner replacement protocols, attachment configuration, and treatment stage, may also explain residual heterogeneity. These variables varied across the 6 included studies and were often incompletely reported, preventing further quantitative assessment. Future primary studies should report these technical and clinical details in a standardized manner to facilitate more comprehensive subgroup analyses in subsequent systematic reviews.

### 4.8. Limitations of the meta-analysis

This meta-analysis has several limitations. First, only 6 retrospective non-randomized studies were included, and their overall risk of bias ranged from low to moderate. Second, individual participant-level data were unavailable, limiting analyses of age, sex, malocclusion severity, patient adherence, attachment design, and programmed expansion magnitude. Third, the included studies differed in anatomical measurement landmarks, measurement software, treatment protocols, follow-up duration, participant age, and outcome definitions. Fourth, the quantitative syntheses and subgroup analyses were based on small numbers of studies, and moderate-to-substantial heterogeneity was present for several outcomes, particularly the canine region. The country-based subgroup analysis was exploratory and may be affected by ecological bias and unmeasured study-level differences. Egger regression test also had limited statistical power because only 6 studies were available for each outcome. These limitations may have reduced the precision and certainty of the pooled estimates.

Given these limitations, the findings should be interpreted cautiously. Future studies should use prospective designs, larger sample sizes, standardized measurement methods, clearly reported treatment protocols, and consistent definitions of predictability. Well-designed controlled clinical studies are needed to confirm the findings of this meta-analysis.

## 5. Conclusion

The available evidence suggests that Invisalign has variable predictability for maxillary transverse expansion across anatomical regions. The highest pooled estimates were observed in the 1st and 2nd premolar regions, whereas lower estimates were found in the canine and 1st molar regions. The canine estimate should be interpreted particularly cautiously because of substantial between-study heterogeneity and low-certainty evidence. Exploratory subgroup analyses indicated that anatomical measurement landmarks, measurement software, and study-level country classification may be associated with differences in reported predictability; however, the small number of studies precludes causal interpretation.

The limited number of retrospective studies, methodological variability, and risk of bias preclude firm conclusions. Future prospective, multicenter studies with larger samples, standardized anatomical landmarks, clearly reported aligner and attachment protocols, and consistent definitions of predictability are needed to confirm these findings and inform individualized clinical planning.

## Acknowledgments

This study was supported by the Scientific Research Program of Hunan Provincial Health Commission (Grant No. W20243152).

## Author contributions

**Conceptualization:** Hou XiangGang, Cao Yani, Jincai Guo.

**Data curation:** Hou XiangGang, Qin Jia, Yang Jie, Cao Yani, Liu Chao, Jincai Guo.

**Formal analysis:** Jincai Guo, Minghai Bai.

**Funding acquisition:** Minghai Bai.

**Investigation:** Qin Jia, Yang Jie, Cao Yani, Jincai Guo, Minghai Bai.

**Methodology:** Hou XiangGang, Jincai Guo, Minghai Bai.

**Project administration:** Minghai Bai.

**Resources:** Minghai Bai.

**Software:** Hou XiangGang, Qin Jia, Yang Jie, Cao Yani, Liu Chao.

**Supervision:** Liu Chao, Jincai Guo, Minghai Bai.

**Validation:** Hou XiangGang, Qin Jia, Yang Jie, Cao Yani, Liu Chao, Jincai Guo.

**Visualization:** Hou XiangGang, Yang Jie, Liu Chao.

**Writing – original draft:** Hou XiangGang.

**Writing – review & editing:** Hou XiangGang, Liu Chao, Jincai Guo.
